# The illusion of cell immortality

**DOI:** 10.1054/bjoc.2000.1296

**Published:** 2000-09-04

**Authors:** L Hayflick

**Affiliations:** Department of Anatomy, University of California, San Francisco, School of Medicine, P.O. Box 89, The Sea Ranch, CA 95497, USA

**Keywords:** mortality, immortality, cell senescence, ageing, longevity determination, telomeres, telomerase

## Abstract

Normal cultured cell populations are mortal but cells that are immortal are abnormal and most have properties of cancer cells. Nevertheless, this distinction becomes blurred because the terms ‘mortality’ and ‘immortality’ are subject to enormous variations in understanding. Forty years ago we showed that cell mortality and immortality are inextricably linked to longevity determination, ageing and cancer. We suggested that a counting mechanism existed in normal cells and that has now been identified as telomere attrition. This replicometer, in combination with the discovery of the enzyme telomerase, has gone very far in explaining why most normal somatic cells have a finite capacity to replicate both in vivo and in vitro and how immortal cancer cells circumvent this inevitability. It is suggested that telomere attrition may be better understood as a direct measure of longevity determination and to only have an indirect association with age changes. © 2000 Cancer Research Campaign


*British Journal of Cancer* (2000) **83**(7), 841–846
© 2000 Cancer Research Campaign
doi: 10.1054/ bjoc.2000.1296, available online at http://www.idealibrary.com on IDEAL®

Millennium mini-review

### The illusion of cell immortality

**L Hayflick**

Department of Anatomy, University of California, San Francisco, School of Medicine, P.O. Box 89, The Sea Ranch, CA 95497, USA

**Summary** Normal cultured cell populations are mortal but cells that are immortal are abnormal and most have properties of cancer cells. Nevertheless, this distinction becomes blurred because the terms 'mortality' and 'immortality' are subject to enormous variations in understanding. Forty years ago we showed that cell mortality and immortality are inextricably linked to longevity determination, ageing and cancer. We suggested that a counting mechanism existed in normal cells and that has now been identified as telomere attrition. This replicometer, in combination with the discovery of the enzyme telomerase, has gone very far in explaining why most normal somatic cells have a finite capacity to replicate both in vivo and in vitro and how immortal cancer cells circumvent this inevitability. It is suggested that telomere attrition may be better understood as a direct measure of longevity determination and to only have an indirect association with age changes. © 2000 Cancer Research Campaign

**Keywords**: mortality; immortality; cell senescence; ageing; longevity determination; telomeres; telomerase

Most cancer cell populations can be distinguished from normal cells by a progressive acquisition of properties that includes immortality. This was first demonstrated in the early part of the twentieth century by the finding that rodent tumours could be serially transplanted indefinitely (Foulds, 1969). By mid century the immortality of cancer cells was demonstrated in vitro (Earle, 1943), followed by the finding that, contrary to prevailing belief, cultured normal cells are mortal (Hayflick and Moorhead, 1961; Hayflick, 1965). We suggested that the finite capacity of normal cells to replicate was a reflection of ageing or longevity determination at the cell level, an idea that received substantial support in subsequent years (Hayflick, 1997, 1998a).

Nevertheless, this distinction between normal and cancer cells has become blurred because the terms 'mortality' and 'immortality' are subject to enormous variations in understanding.

In the legal sense the mortality of a human is established in many countries when a physician certifies that brain death has occurred. However, acceptable evidence of death varies among different legal systems. Immortality, on the other hand, was rarely considered as a legal concept until it became possible in 1980 to patent self-reproducing life forms (Hayflick, 1998b).

Where certification of human brain death is a prerequisite for the legal removal of donor tissue, the expectation is that the remaining organs, tissues or cells that may be taken for transplantation are viable. Thus, legally, the mortality of one organ – the brain – is assumed to have no immediate effect on the mortality and subsequent usefulness of the remaining organs.

The determination of mortality and immortality is even more complex in a stricter biological sense. A common example given for biological immortality is the alleged indefinite replicative capacity of unicellular organisms because cadavers do not result from fission replication and an endless lineage of viable cells are produced. However, this example fails to distinguish between individual and population mortality and immortality. Individual micro-organisms, like individual humans are mortal, but as populations both appear to be immortal. The 'cadavers' of organisms that divide by fission are their molecules and atoms that become increasingly diluted and ultimately lost in those cells at the leading edge of the expanding population. Or, these molecules are lost to further progeny when fission products die.

Most micro-organisms, like higher life forms, are now known to possess mechanisms for genetic recombination (Maynard Smith et al, 1993). In populations of unicellular forms, a narrow lineage may appear to reproduce endlessly but for the same reason that a fraction of the population of higher organisms, like humans, appears to reproduce endlessly. Thus, the finality of life in individual unicellular forms may be no different from mortality in multicellular forms. But, populations of each are immortal, or potentially so, and for the same reasons.

### DEFINITIONS

The most stringent definition of immortality is a life form capable of indefinite survival in conditions where no changes have occurred in molecular composition from some arbitrary beginning. However, this condition is incompatible with life, if for no other reasons than it precludes metabolism and replication.

The only condition that comes close to meeting this definition is cryogenic preservation where cells appear to be capable of indefinite survival at temperatures that approach absolute zero. Under these conditions life processes are arrested but life is not being lived in the sense that metabolic activity is radically reduced or does not occur at all.

*Received* 19 April 2000
*Accepted* 26 April 2000

*Correspondence to:* L Hayflick

My normal human diploid cell strain WI-38 (Hayflick, 1965) has been cryogenically preserved in a viable state for over 38 years. This is the longest period of time that a viable normal human cell population has ever been preserved. Absent failure to top up the liquid nitrogen freezer periodically, these cells can be considered to be immortal. But, these cells are not immortal in the sense that metabolism has continued uninterrupted during the past 38 years.

A more liberal definition of biological immortality would be the indefinite survival of a life form whose vital life processes function indefinitely. Yet, this definition is not without difficulty because the quality of molecular 'sameness' is lost. This concept of molecular identity is best illustrated by challenging a tenet of modern biology.

In 1891 August Weismann proposed that the germ plasm is immortal. Yet, the repeated replication and repair of DNA results in molecules that do not contain atoms of the same nucleotides present in the DNA of a founding cell. Also, different genes are contributed from each parent at every fusion that further contributes to loss of sameness. Finally, given equal distribution at each division, other molecules or atoms present in a founding cell are not likely to be present after the clone has produced a lineage of about fifty population doublings.

The reasoning is that $3.3 \times 10^9$ molecules of $H_2$ are roughly equivalent to the mass of a human fibroblast so that the first cell in the lineage must consist entirely of $H_2$ to permit one molecule to reach each cell at the 50th doubling (Hayflick and Moorhead, 1961). This assumes equal distribution of molecules at each division. Because cells are not composed entirely of the smallest molecule and because molecular turnover and repair occur, the likelihood is that those cells present well before the lineage reaches 50 population doublings retain few, if any, of the molecules present in the founding cell.

Thus, there is a tenable argument that the germ plasm is not immortal because molecules at the end of a lineage of zygotes are not identical to those in the founding gametes. A closer approximation to fidelity is that each zygote in the lineage contains the same genetic information as does its precursors following fertilization. This is dubious because of the occurrence of mutations, one of the few universal properties of life.

Cells at the leading edge of any long lineage do not have the identical molecular content as existed in the founding cell. Even in non-dividing cells, molecular turnover must occur (Lindahl, 1993) leading to the conclusion that, like dividing cells, non-dividing cells also are not molecularly identical after the passage of time.

Thus, August Weismann may have been wrong not only because molecular turnover is inevitable in all cell lineages but because the alleged immortality of the germ plasm takes place only under conditions of periodic genetic recombination. By themselves gametes and the germ plasm are not an immortal lineage.

### MORTALITY AND AGEING

Another example of the difficulty of defining mortality impinges on our concept of age. For example, there is a common belief that Redwood trees and Bristle cone pines can achieve great age.

Yet, the cells in these trees, alleged to be thousands of years old, are dead. The living cells in the needles, cambium layer and root hairs are no older than about 30 years (Hayflick, 1996). Humans, older than 30 years, who stand in awe of Redwood trees should more accurately stand in awe of their own living post mitotic neurons that are older than the tree's living cells.

The quaking aspen clones estimated to be over 10 000 years old (Cooke, 1985), creosote bushes (*Larrea tridentate*) said to be 11 700 years old (Vasek 1980), and the 1400 year-old bracken ferns (*Pteridium aquilinum*) (Oinenen, 1967) all suffer from the same criticism. Finally, the sea anemone, frequently cited as an example of an organism hundreds of years old (Hughes, 1989), if not immortal, is a colonial animal whose component cells turnover regularly thus making an argument for their immortality, or great longevity, spurious.

Animals that do not reach a fixed size in adulthood (examples include the American lobster, some reptilians, many sport and deep sea fish) either do not age, or the process is so slow that the rate is imperceptible. They are not immortal because, like animals that do age, there is an annual likelihood of death attributable to disease, accident or predation (Hayflick, 1996).

These examples represent the difficulty in defining biological mortality and immortality that can become serious impediments to understanding and accurate communication.

In its simplest, though still inaccurate form, biological mortality is usually defined as the death of an organism or the termination of its lineage. Immortality would be defined as the indefinite survival of a single organism or of a replicating population regardless of molecular turnover. Imperfect as these definitions are, they do have limited use in understanding some of the fundamental differences between normal and cancer cells.

### IMMORTAL CANCER CELLS

One of the striking characteristics of cultured cancer cell lines is the property of population immortality.

This distinction first became apparent to us about 40 years ago when we observed that, contrary to the prevailing belief that all cultured cells were potentially immortal, cultured normal cells have a limited capacity for replication (Hayflick and Moorhead, 1961). Because we knew that several populations of apparently immortal cancer cell populations existed – best exemplified by HeLa – we proposed that normal cells are mortal and that immortal cell populations had acquired cancer cell properties (Hayflick and Moorhead, 1961; Hayflick, 1965).

Our results led us to conclude that these two classes of cultured cells bear the following relationship to their in vivo counterparts (Hayflick 1965):

| Heteroploid cell lines | : | Transplantable tumours | = | Diploid cell strains | : | Normal somatic tissue |
|---|---|---|---|---|---|---|
| (in vitro) | | (in vivo) | | (in vitro) | | (in vivo) |
| 1. Heteroploid | | | | 1. Diploid | | |
| 2. Cancer cells | | | | 2. Normal cells | | |
| 3. Indefinite growth | | | | 3. Finite growth | | |

*© 2000 Cancer Research Campaign*

This was the first effort to classify cell populations based on the properties of mortality or immortality. Had it not been shown that normal cells are mortal, the subsequent interest in mechanisms that lead to immortalization would not have been appreciated. The acquisition of the property of immortality by normal cells currently is a fundamental underpinning of both aging and cancer research. In the following discussion the terms “cell line” and “cell strain” will be used as defined above.

The acquisition by a cell strain of the properties described above for a cell line is defined here as ‘transformation’.

### IMMORTALITY DISPROVED

In protozoa where an individual is isolated and continuously separated from daughter cells as they are produced (‘isolate’ cultures), mortal lineages always occur, thus settling a controversy of 50 years duration (Bell, 1988). Alleged ‘immortal’ cell lines, like HeLa, that have been cultivated continuously for decades, but never studied as isolate cultures, cannot be assumed to be immortal.

Isolate cultivation of cell lines could reveal that immortalization, a major characteristic of transformation, may be only indirectly dependent upon interventions using radiation, chemical carcinogens, oncogenic viruses like SV40, transfection with particular oncogenes like c-myc or activation of normal cellular proto-oncogenes.

These procedures may confer upon mortal cells the ability to periodically undergo sexual recombination that may be the ultimate prerequisite for population immortality in cell lines. Like unicellular populations of protozoa or micro-organisms, cell lines may achieve population immortality only after an accumulated load of deleterious mutations (Muller’s Ratchet) has been disposed of by sexual recombination (mixis). To ‘immortalize’ a normal cell population may mean to induce some cells in the population to acquire the ability to undergo mixis. Somatic cell genetic recombination is predicted from this reasoning and would be a most important finding.

The appearance and disappearance of gap junctions or intercellular channels known as connexins which link cells to each other provide important opportunities to explore the possibility that genetic information may flow between somatic cells (Paul and White, 1999).

Like the myth of Alexis Carrel’s immortal chick heart fibroblasts (Witkowski 1979, 1980, 1985), the 50-year-old assumption that cell lines are immortal may also be a myth because the occurrence of mixis has not been disproved. A more recent assumption of immortality has been made for cultured embryonic stem cells, yet proof that these cells can be propagated continuously for years with retention of normal properties, even in the absence of genetic recombination, has yet to been demonstrated.

### MECHANISMS OF IMMORTALIZATION

Continuously replicating cell lines may arise spontaneously from normal cell strains by unknown processes or created in the laboratory as described above.

It has been recognized for many years that there is great species variability in the capacity of cells to spontaneously transform (Macieira-Coelho, 1999). Least likely are human and chicken cells and the easiest are rodent cells but even they do not express a frequency of occurrence greater than one in a million (Kraemer et al, 1986). There is no good explanation for these differences. A possibility that has not been excluded might be that cells of species most likely to transform in vitro are derived from highly inbred laboratory animals.

Hybridization studies between human mortal and immortal populations reveal that immortality is a recessive trait (Ran and Periera-Smith, 2000). The opposite is true for rodent cells where immortality appears to be a dominant trait. Immortal murine hybridomas can be obtained from the fusion product of immortal myeloma cells with mortal antibody-producing lymphocytes (Kohler and Milstein, 1975), a property of murine cells that also may be linked to the relative ease with which they spontaneously transform.

The process of immortalization appears to be one of the earliest events in the stepwise progression necessary for full expression of the transformed phenotype. In Syrian and Chinese hamster, mouse, rat and pig cell immortality is followed by anchorage independence, reduced serum growth factor requirements and finally tumorigenicity (Kraemer et al, 1986). Human cells, however, may reveal tumorigenic properties before they reveal immortality (Sack, 1981).

### THE IDEA OF A COUNTER

Three of our early observations led us to conclude that normal, mortal, human cells must contain a replication counting mechanism. First, was the reproducibility of our finding that human fibroblasts from different embryonic donors underwent a finite number of population doublings that spanned a narrow range between 40 and 60. Second, cells frozen at any population doubling level from 1 to 50 retained memory of that level until reconstitution so that the total number of population doublings traversed both before and after freezing totalled 50 (Hayflick and Moorhead, 1961; Hayflick, 1965). The ability of WI-38 to remember at what population doubling it is when frozen is as accurate today as it was when I first developed the strain in 1962. After 38 years of cryopreservation, WI-38’s memory has been retained without loss.

The third evidence for the existence of a counter resulted from our efforts in 1975 to determine the location of the putative counter. By employing enucleation techniques in which the nuclei of old and young cultured cells were fused to opposite aged enucleated cytoplasts we concluded that the replicometer was located in the nucleus (Wright and Hayflick, 1975; Muggleton-Harris and Hayflick, 1976).

The search for the counting mechanism remained virtually quiescent for the next 15 years. Then, the remarkable convergence of data from several diverse fields of research with our own resulted in an explosion of fascinating information that has not only determined the location of the counting mechanism but has identified its molecular structure. (For more detailed reviews see Blackburn and Greider, 1995; Kipling, 1995; Chiu and Harley, 1997; Greider, 1998; Hayflick, 1997.)

### THE TELOMERE REPLICOMETER

As suggested cell mortality and immortality are inextricably linked to ageing and cancer (Hayflick, 1965). Consequently, the importance of identifying the putative counter would be difficult to exaggerate.

© 2000 Cancer Research Campaign

The counting mechanism should not be called a clock or chronometer because these devices measure the passage of time. The replicative limit of normal cells is directly related to the number of cell doublings, or more precisely DNA replications. Thus, the putative mechanism should be more properly referred to as an event counter. Because a meter is a device that measures quantity, or counts events, this would justify the suggestion that the term 'replicometer' be used in lieu of 'clock'.

In a 1938 lecture given by Hermann Muller (1962) and followed by the work of Barbara McClintock (1941), the tips of chromosomes were reported to contain discrete structures called telomeres. Although the precise role that these structures played was unclear, there was some evidence that telomeres prevented chromosomes from fusing to each other end to end and that they permitted the attachment of chromosome ends to the nuclear envelope.

In an entirely different area of biological inquiry it was observed, in the early 1970s, that the properties of DNA polymerase prevent it from fully replicating the linear ends of DNA (Olovnikov, 1971; 1973; 1996; Watson, 1972). This 'end-replication problem' is the inability of DNA polymerase to completely replicate the 3′ end of linear duplex DNA.

In the late 1960s, Alexey Olovnikov, who had just heard a lecture on our work, wondered how normal cells might have a limited capacity to replicate as he entered a Moscow subway station (Olovnikov, 1996). When the train stopped at the station he had a remarkable flash of insight. Olovnikov saw an analogy between the train, which represented the DNA polymerase, and the track that represented the DNA. If the train engine were imagined to be the polymerase that replicated the DNA track, the first segment of DNA would not be replicated because it was underneath the engine at the start. This was analogous to the 'end-replication problem'. Olovnikov realized that this repeated shortening of the DNA molecule at each round of DNA replication might explain our finding that normal cells can only replicate a specific number of times. James Watson independently arrived at a similar solution in 1972.

Because the loss of DNA that contained vital genetic information at each division seemed unlikely, Olovnikov reasoned that telomeres might consist of repeated nucleotide sequences that did not contain genetic information but behaved much like a buffer. At each round of DNA replication the buffer would simply lose what portion of the DNA molecule was not copied (the telomeric ends) and thus protect downstream genes. The length of the buffer would determine the number of rounds possible for DNA replication.

Olovnikov's imaginative solution to the end-replication problem, although published in 1971 in Russian and in 1973 in English, languished in the literature for several years until laboratory reports emerged that have substantially supported his armchair speculations.

### TELOMERE STRUCTURE DISCOVERED

In 1978, Elizabeth Blackburn, working with the ciliated protozoan, *Tetrahymena*, found that telomeres consisted of a simple sequence of hexameric repeats of the nucleotides TTGGGG (Blackburn and Gall, 1978). It was later found that the telomere repeat sequence in human cells was TTAGGG (Moyzis et al, 1988). From slime moulds to humans, telomeres consist of thousands of repeats of the highly conserved sequence TTAGGG (Henderson, 1995).

In 1986 Howard Cooke suggested that telomere shortening might occur in human cells (Cooke and Smith, 1986). Later Calvin Harley, who had worked for several years with my system of senescent human cells, had a fortuitous discussion with Carol Greider and both decided to explore the possibility that the limited proliferative capacity of cultured normal cells might be explained by diminishing telomere length. They did, indeed, find that the mean telomere length decreased by 2 to 3 kilobase pairs during the entire in vitro lifetime of several strains of cultured normal human diploid fibroblasts (Harley et al, 1990).

The decrease was found to be progressive and averaged 50 base pairs per population doubling (Levy et al, 1992). The telomere shortening seen in ageing cultured normal human fibroblasts also occurs in many other normal cultured and in vivo cell types.

Allsopp et al (1992) reported that after analysing the cultured normal fibroblasts from 31 human donors, aged from several months to 93 years, a striking correlation, valid over the entire age range, was found between replicative capacity and initial telomere length. Thus, cell strains with shorter telomeres underwent significantly fewer doublings than those with longer telomeres.

Telomeric shortening appears to be the replicometer that determines the number of times that a normal cell is able to divide. Once a threshold number of telomeric $(TTAGGG)_n$ repeats is reached, downstream events presumably are triggered that signal the cessation of DNA replication. Wright and Shay (1992) have offered an alternative explanation of how telomere shortening acts as a replicometer. Their telomere positional effect explanation of cell senescence is based on a novel two-stage model.

### ACHIEVING IMMORTALITY

One essential question remains: How do immortal cell lines avoid telomere shortening that, if it occurs, would lead to their demise?

The answer to this critical question also originated in studies with *Tetrahymena* by Greider and Blackburn (1985), who discovered the ribonucleoprotein enzyme terminal transferase called telomerase. They found that telomeres are synthesized de novo by telomerase, a ribonucleoprotein enzyme that extends the 3′ end of telomeres and thus elongates them. This ribonucleoprotein complex contains a reverse transcriptase and RNA template for the synthesis of the repeated sequence (Shippen-Lentz and Blackburn, 1990). It was simultaneously reported that cancer cells have shorter telomeres than do adjacent normal cells (de Lange et al, 1990; Hastie et al, 1990) thus providing the first link for the role of telomeres in cancer biology.

Telomerase was later found to occur in extracts of immortal human cell lines (Morin, 1989; Counter et al, 1992) and in about 90% of all human tumours studied (Chiu and Harley, 1997). Subsequently, the telomerase RNA component (Feng et al, 1995) and the catalytic portion of the enzyme were cloned (Nakamura et al, 1997). Telomerase is the only known reverse transcriptase that is necessary for normal cell activity.

Unlike normal mortal cultured cell strains, most immortal cell lines produce telomerase. Thus, the telomeres of most cell lines do not shorten with serial passage in vitro.

In recent years telomerase expression has been found in several classes of normal cells. These include fetal tissue, normal bone marrow stem cells, testes, peripheral blood lymphocytes, skin epidermis and intestinal crypt cells (Chiu and Harley, 1997). Prior to the 10th post-natal week in humans the RNA component of

*© 2000 Cancer Research Campaign*

telomerase can be found in virtually all differentiating cells (Yashima et al, 1998). All of these normal cells have high turnover rates or are in a continuously replicating pool of differentiating cells. The level of telomerase activity found in normal cell populations is, per cell, significantly less than that found in cancer cells (Chiu and Harley, 1997).

The observation of telomere attrition as normal cells divide, provides the first direct evidence for the putative replicometer. This, in combination with the discovery of the enzyme telomerase, has gone very far in explaining why most normal somatic cells have a finite capacity to replicate in vivo and in vitro and how immortal cancer cells circumvent this inevitability.

In 1998 it was reported that normal, mortal, human cell strains could be immortalized with apparent retention of their normal properties by transfecting them with vectors encoding the human telomerase catalytic subunit (Bodnar et al, 1998). Thus, the replicometer can be purposefully circumvented. This has provided direct evidence proving the role of telomere shortening in cell senescence and telomerase expression in cell immortality.

This discovery has profound theoretical and practical implications that include the immortalization of highly differentiated normal human cell types for the production of medically useful proteins.

Because exquisitely sensitive methods exist for the detection of telomerase in a single cell, this procedure is being exploited as a diagnostic tool to detect the presence of cancer cells in clinical specimens. Other researchers are exploring the possibility that telomerase inhibitors might be found that could be used therapeutically in the treatment of cancer (de Lange and Jacks, 1999; Zhang et al, 1999).

An intriguing possibility that has not yet been explored is the role that telomerase might play in the induction of genetic recombination which, as indicated earlier, might be a prerequisite for immortalization.

As indicated earlier, animals that do not reach a fixed size in adulthood either do not age or the rate is undetectable. Fascinating recent findings reveal that in the rainbow trout (*Oncorhynchus mykiss*), an animal with imperceptible age changes, and unlike animals that do age, high telomerase activity has been found in the cells of all of the organs analysed (Klapper et al, 1998).

### TELOMERE ATTRITION AS A LONGEVITY DETERMINATOR

Telomere attrition may be better understood as a measure of longevity determination than it might be as a cause of age changes. After reproductive maturity in animals that age, the level of remaining physiological reserve determines longevity. The reserve does not renew at the same rate that it incurs losses because molecular disorder increases at a rate greater than does capacity for repair. This increase in molecular disorder is aging which increases vulnerability to predation, accidents or disease (Hayflick, 1998a).

Hundreds of biological changes occur in normal cells as they replicate in vitro (Hayflick, 1980) resulting in increasing molecular disorder and loss of cell function. Thus, the number of population doublings that a cell strain is capable of undergoing and that is determined by telomere length, may be the in vitro expression of maximum potential longevity. The hundreds of molecular disorders that herald the approaching loss of replicative capacity, and diminution of telomere length, are age changes. When molecular disorder occurs in dividing cells in vivo, post mitotics may also be affected and both may then reveal increasing vulnerability to pathology and subsequent death of the individual well before species maximum longevity is reached (Hayflick, 1996).

### ORDINARY MORTALS

It is probable that biological immortality has been a part of human thinking since the first humans realized their own mortality. Immortality, as a concept, appeared in writings at least 3500 years ago, was a major goal in alchemy, and in the last half of this millennium drove vast explorations in search of the fountain of youth (Hayflick, 1996). In today's iteration, the pursuit of great longevity is driven by the belief that a particular diet, life style or concoction will achieve the desired result.

The serious problems that could occur if immortality or extreme longevity was to become possible have been the theme of much of our literature, starting with Greek mythology in which the Trojan Tithonus loves Eos, the Goddess of dawn. At her request, Zeus makes Tithonus immortal but, unfortunately, Eos neglects to also ask that he not age. Jonathan Swift rediscovered this theme in his immortal, but continuously aging, Struldbrugs.

The fact that immortality, in its stricter definitions has never been demonstrated, even in unicellular forms, provides strong support for the likelihood, if not the hope, that it will not be found to occur in higher forms.

### REFERENCES

Allsopp RC, Vaziri H, Patterson C, Goldstein S, Younglai EV, Futcher AB, Greider CW and Harley CB (1992) Telomere length predicts replicative capacity of human fibroblasts. *Proc Natl Acad Sci* **89**: 10114–10118

Bell G (1988) *Sex and Death in Protozoa*. The History of an Obsession. Cambridge University Press: Cambridge

Blackburn EH and Gall JG (1978) A tandemly repeated sequence at the termini of the extrachromosomal ribosomal RNA genes in Tetrahymena. *J Mol Biol* **120**: 33–53

Blackburn EH and Greider CW (1995) (eds.) *Telomeres*, Cold Spring Harbor Laboratory Press

Bodnar AG, Ouellette M, Frolkis M, Holt SE, Chiu C, Morin GB, Harley CB, Shay JW, Lichsteiner S and Wright WE (1998) Extension of life span by introduction of telomerase into normal human cells. *Science* **279**: 349–352

Chiu C and Harley CB (1997) Replicative senescence and cell immortality: the role of telomeres and telomerase. *Proc Soc Exp Biol Med* 99–106

Cooke HJ and Smith BA (1986) Variability of the telomeres of the X/Y pseudoautosomal region. *Cold Spring Harbor Symp. Quant Biol* **51**: 213–219

Cooke RE (1985) Growth and development in clonal plant populations. In: Jackson JBC, Buss LW and Cooke RE (eds.) *Population Biology and Evolution of Clonal Organisms*, Yale University Press, New Haven, Conn, pp 259–296

Counter CM, Avilon AA, LeFeuvre CE, Stewert MG, Greider CW, Harley CB and Bacchetti S (1992) Telomere shortening associated with chromosome instability is arrested in immortal cells which express telomerase activity. *EMBO J* **11**: 1921–1929

de Lange T and Jacks T (1999) For better or worse? Telomerase inhibition and cancer. *Cell* **98**: 273–275

de Lange T, Shiue L, Myers MR, Cox DR, Naylor SL, Killery AM and Varmus HE (1990) Structure and variability of human chromosome ends. *Mol Cell Biol* **10**: 518–527

Earle WR (1943) Production of malignancy in vitro. *J Natl Cancer Inst* **4**: 165–172

Feng J, Funk WD, Wang S-S, Weinrich SL, Avilion AA, Chiu CP, Adams RR, Chang E, Allsopp RC, Yu J, Le S, West MD, Harley CB, Andrews WH, Greider CW and Villeponteau B (1995) The RNA component of human telomerase. *Science* **269**: 1236–1241

Foulds L (1969) *Neoplastic Development I.* Academic Press, London, New York

Greider CW (1998) Telomeres and senescence: The history, the experiment, the future. *Current Biology* **8**: 178–181

© 2000 Cancer Research Campaign

Greider CW and Blackburn EH (1985) Identification of a specific telomere terminal transferase enzyme with two kinds of primer specificity. *Cell* **51**: 405–413

Harley CB, Futcher AB and Greider CW (1990) Telomeres shorten during ageing of human fibroblasts. *Nature* **345**: 458–460

Hastie ND, Dempster M, Dunlop MG, Thompson AM, Green DK and Allshire RC (1990) Telomere reduction in human colorectal carcinoma and with ageing. *Nature* **346**: 866–868

Hayflick L (1965) The limited in vitro lifetime of human diploid cell strains. *Exp Cell Res* **37**: 614–636

Hayflick L (1980) Cell Aging. In: *Annual Review of Gerontology and Geriatrics*, C. Eisdorfer (ed), Springer Publishing Co., NYC Vol. I, 26–67

Hayflick L (1996) *How and Why We Age*, Ballantine Books, New York City, N.Y.

Hayflick L (1997) Mortality and immortality at the cellular level: A review. *Biochemistry (Moscow)* **62**: 1180–1190

Hayflick L (1998a) How and why we age. *Exp Gerontol* **33**: 639–653

Hayflick L (1998b) A novel technique for transforming the theft of mortal human cells into praiseworthy federal policy. *Exp Gerontol* **33**: 191–207

Hayflick L and Moorhead PS (1961) The serial cultivation of human diploid cell strains. *Exp Cell Res* **25**: 585–621

Henderson E (1995) Telomere DNA structure. In: *Telomeres*, Blackburn EH and Greider CW (eds.) Cold Spring Harbor Laboratory Press, pp. 11–34

Hughes RN (1989) *A Functional Biology of Clonal Animals*, Chapman and Hall, NY p. 169.

Kipling D (1995) *The Telomere*, Oxford University Press

Klapper W, Heidorn K, Kühne K, Parwaresch R and Krupp G (1998) Telomerase in ‘immortal’ fish, *FEBS Letters*. **434**: 409–412

Kohler G and Milstein C (1975) Continuous cultures of fused cells secreting antibody of predefined specificity. *Nature* **256**: 495–497

Kraemer PM, Ray FA, Brothman AR, Bartholdi MF and Cram LS (1986) Spontaneous immortalization rate of cultured Chinese hamster cells. *J Natl Cancer Inst* **76**: 703–709

Levy MZ, Allsopp RC, Futcher AB, Grieder CW and Harley CB (1992) Telomere end-replication problem and cellular aging. *J Molec Biol* **225**: 951–960

Lindahl T (1993) Instability and decay of the primary structure of DNA. *Nature* **362**: 709–715

Macieira-Coelho A (1999) A comparative biology of cell immortalization. In *Cell Immortalization*, Macieira-Coelho A (ed.) 51–80, Springer-Verlag: Heidelberg

Maynard Smith J, Smith NH, O’Rourke M, Spratt BG (1993) How clonal are bacteria? *Proc Natl Acad Sci USA* **90**: 4384–4388

McClintock B (1941) The stability of broken ends of chromosomes in *Zea mays*. *Genetics* **26**: 234–282

Morin GB (1989) The human telomere terminal transferase enzyme is a ribonucleoprotein that synthesizes TTAGGG repeats. *Cell* **59**: 521–529

Moyzis RK, Buckingham JM, Cram LS, Dani M, Deaven LL, Jones MD, Meyne J, Ratliff RL and Wu J-R (1988) A highly conserved repetitive DNA sequence (TTAGGG)n, present at the telomeres of human chromosomes. *Proc Natl Acad Sci* **85**: 6622–6626

Muggleton-Harris AL and Hayflick L (1976) Cellular aging studied by the reconstruction of replicating cells from nuclei and cytoplasms isolated from normal human diploid cells. *Exp Cell Res* **103**: 321–330

Muller HJ (1962) The remaking of chromosomes. In: *Studies of Genetics: The Selected Papers of H.J. Muller*, pp 384–408. Indiana University Press, Bloomington

Nakamura TM, Morin GB, Chapman KB, Weinrich SL, Andrews WH, Lingner J, Harley CB and Cech TR (1997) Telomerase catalytic subunit homologs from fission yeast and humans. *Science* **277**: 955–959

Oinenen E (1967) The correlation between the size of Finnish bracken (*Pteridium aquilinum* [L.] Kuhn.) clones and certain periods of site history. *Acta Forrestalia Fennica* **83**: 1–51

Olovnikov AM (1971) Principles of marginotomy in template synthesis of polynucleotides. *Dokl Akad Nauk S S S R* **201**: 1496–1499

Olovnikov AM (1973) A theory of marginotomy: The incomplete copying of template margin in enzyme synthesis of polynucleotides and biological significance of the problem, *J Theoret Biol* **41**: 181–190

Olovnikov AM (1996) Telomeres, telomerase and aging: origin of the theory. *Exp Geront* **31**: 443–448

Paul T and White D (1999) Genetic diseases and gene knockouts reveal diverse connexin functions. *Ann Rev Physiol* **61**: 283–310

Ran Q and Periera-Smith OM (2000) Genetic approaches to the study of replicative senescence. *Exp Gerontol* **35**: 7–13

Sack GH (1981) Human cell transformation by simian virus 40. A review. *In vitro* **17**: 1–19

Shippen-Lentz D and Blackburn EH (1990) Functional evidence for an RNA template in telomerase. *Science* **247**: 546–552

Vasek FC (1980) Creosote bush: long-lived clones in the Mohave Desert. *Am J Botany* **67**: 246–255

Watson JD (1972) Origin of concatemeric T7 DNA. *Nature, New Biol* **239**: 197–201

Weismann A (1891) *Essay Upon Heredity and Kindred Biological Problems*, 2nd ed. Clarendon Press, Oxford

Witkowski JA (1979) Alexis Carrel and the mysticism of tissue culture. *Medical History* **23**: 279–296

Witkowski JA (1980) Dr. Carrel’s immortal cells. *Medical History* **24**: 129–142

Witkowski JA (1985) The myth of cell immortality. *Trends in Biochemical Sciences* **10**: 258–260

Wright WE and Hayflick L (1975) Nuclear control of cellular aging demonstrated by hybridization of anucleate and whole cultured normal human fibroblasts. *Exp Cell Res* **96**: 113–121

Wright WE and Shay JW (1992) Telomere positional effect and the regulation of cellular senescence. *Trends in Genetics* **8**: 193–197

Yashima K, Maitra A, Rogers BB, Timmons CF, Rathi A, Pinar H, Wright WE, Shay JW and Gazdar AF (1998) *Cell Growth and Differentiation* **9**: 805–813

Zhang X, Mar V, Zhou W, Harrington L and Murray RO (1999) Telomere Shortening and apoptosis in telomerase-inhibited human tumor cells. *Genes and Development* **13**: 2388–2399

 *© 2000 Cancer Research Campaign*

